# Peptidyl Prolyl Isomerase PIN1 Directly Binds to and Stabilizes Hypoxia-Inducible Factor-1α

**DOI:** 10.1371/journal.pone.0147038

**Published:** 2016-01-19

**Authors:** Hyeong-jun Han, Nayoung Kwon, Min-A Choi, Kyung Oh Jung, Juan-Yu Piao, Hoang Kieu Chi Ngo, Su-Jung Kim, Do-Hee Kim, June-Key Chung, Young-Nam Cha, Hyewon Youn, Bu Young Choi, Sang-Hyun Min, Young-Joon Surh

**Affiliations:** 1 Tumor Microenvironment Global Core Research Center, College of Pharmacy, Seoul National University, Seoul, 151–742, South Korea; 2 Research Institute of Pharmaceutical Sciences, College of Pharmacy, Seoul National University, Seoul, 151–742, South Korea; 3 Department of Nuclear Medicine, Seoul National University, Seoul, 136–742, South Korea; 4 Biomedical Sciences, Seoul National University, Seoul, 136–742, South Korea; 5 Cancer Research Institute, Seoul National University, Seoul, 136–742, South Korea; 6 Department of Molecular Medicine and Biopharmaceutical Sciences, Graduate School of Convergence Science and Technology, Seoul National University, Seoul, 151–742, South Korea; 7 College of Medicine, Inha University, Incheon, South Korea; 8 Cancer Imaging Center, Seoul National University Hospital, Seoul, 136–742, South Korea; 9 Department of Pharmaceutical Science and Engineering, School of Convergence Bioscience and Technology, Seowon University, Cheongju, 361–742, South Korea; 10 New Drug Development Center DGMIF, Daegu, 701–310, South Korea; Duke University Medical Center, UNITED STATES

## Abstract

Peptidyl prolyl isomerase (PIN1) regulates the functional activity of a subset of phosphoproteins through binding to phosphorylated Ser/Thr-Pro motifs and subsequently isomerization of the phosphorylated bonds. Interestingly, PIN1 is overexpressed in many types of malignancies including breast, prostate, lung and colon cancers. However, its oncogenic functions have not been fully elucidated. Here, we report that PIN1 directly interacts with hypoxia-inducible factor (HIF)-1α in human colon cancer (HCT116) cells. PIN1 binding to HIF-1α occurred in a phosphorylation-dependent manner. We also found that PIN1 interacted with HIF-1α at both exogenous and endogenous levels. Notably, PIN1 binding stabilized the HIF-1α protein, given that their levels were significantly increased under hypoxic conditions. The stabilization of HIF-1α resulted in increased transcriptional activity, consequently upregulating expression of vascular endothelial growth factor, a major contributor to angiogenesis. Silencing of PIN1 or pharmacologic inhibition of its activity abrogated the angiogenesis. By utilizing a bioluminescence imaging technique, we were able to demonstrate that PIN1 inhibition dramatically reduced the tumor volume in a subcutaneous mouse xenograft model and angiogenesis as well as hypoxia-induced transcriptional activity of HIF-1α. These results suggest that PIN1 interacting with HIF-1α is a potential cancer chemopreventive and therapeutic target.

## Introduction

Hypoxia, which results from an imbalance between the supply and use of oxygen in tumor microenvironment, contributes to tumor propagation, malignant progression, and resistance to anticancer therapy [1]. Transcription of many hypoxic-inducible genes is mainly controlled by hypoxia-inducible factor (HIF)-1. These include those genes encoding angiogenic cytokines, such as vascular endothelial growth factor (VEGF) and its receptors VEGFR1 and VEGFR2 [2]. VEGF triggers signal transduction essential for angiogenesis and hence tumor growth [3]. HIF-1 is a heterodimeric protein consisting of HIF-1α and HIF-1β subunits, which are basic helix-loop-helix-PAS domain proteins [4]. HIF-1α accumulates rapidly in cells challenged with hypoxia [5]. Under normoxic conditions, HIF-1α undergoes hydroxylation by prolyl hydroxylase, and subsequently interacts with the von Hippel Lindau (pVHL) protein. This facilitates the HIF-1α degradation through the ubiquitin-proteasome pathway [6]. In hypoxia, however, limited hydroxylation leads to stabilization and accumulation of HIF-1α [7]. Phosphorylation of HIF-1α, among various post-translational modifications, occurs predominantly during hypoxic conditions [8], which influences stability of HIF-1α and its transcriptional activity [9]. The site of phosphorylation is critical for determining the stability of HIF-1α. Polo-like kinase 3 phosphorylates HIF-1α directly on Ser^576^ and Ser^657^ and negatively regulates the stabilization of HIF-1α [10]. In addition, glycogen synthase kinase 3β phosphorylates HIF-1α on Ser^551^, Ser^555^, and Ser^589^ residues, which facilitates degradation of HIF-1α [11]. In contrast, cyclin-dependent kinase1 promotes stabilization of HIF-1α through phosphorylation of HIF-1α on Ser^668^ under both normoxic and hypoxic conditions [12]. However, it remains poorly understood how phosphorylation of HIF-1α influences the stability of HIF-1α.

Phosphorylation-dependent prolyl isomerization is a critical post-translational regulatory mechanism in intracellular signaling [13]. PIN1, a peptidyl-prolyl *cis*/*trans*-isomerase (PPIase), consists of an N-terminal WW domain and a C-terminal PPIase domain [14]. PIN1 binds and isomerizes specific phosphorylated Ser/Thr-Pro (pSer/Thr-Pro) motifs in a subset of proteins, resulting in their conformational changes [13, 15]. Peptidyl-prolyl isomerization alters the conformation of a target protein, modulating its phosphorylation status, interaction with other proteins, subcellular localization, and stability [13, 16]. PIN1 undergoes phosphorylation on its Ser^16^ residue localized in the center of the pSer/Thr-Pro binding pocket. This abolishes capability of PIN1 to interact with its substrates and may facilitate nuclear export [17]. Overexpression of both PIN1 and HIF-1α is prevalent in many types of human cancer [18, 19]. However, little is known about the role of PIN1 in modulating the transcriptional activity of HIF-1α. Here we report that PIN1 binds to and stabilizes HIF-1α, consequently enhancing the angiogenesis.

## Materials and Methods

### Reagents and antibodies

Dulbecco’s modified Eagle’s medium (DMEM), penicillin/streptomycin mixtures, fetal bovine serum (FBS), TRIzol^®^ were purchased from Gibco BRL (Grand Island, NY, USA). Primary antibodies for PIN1 and green fluorescence protein (GFP) were supplied by Santa Cruz Biotechnology (Santa Cruz, CA, USA). Antibodies against phospho-PIN1 and HA-tag were products of Cell Signaling Technology (Beverly, MA, USA). Antibodies against HIF-1α and VEGF-A were purchased from Novus Biologicals (Littleton, CO, USA). An antibody against HIF-1α and the growth factor-reduced Matrigel were purchased from BD Biosciences (Bedford, MA, USA). Antibodies against CD31 and luciferase were purchased from Abcam (Cambridge, UK). Secondary antibodies were purchased from Zymed Laboratories Inc. (San Francisco, CA, USA). The PIN1 inhibitor, PiB (diethyl-1,3,6,8-tetrahydro-1,3,6,8-tetraoxobenzol-phenanthroline-2,7-diacetate), deferoxamine mesylate (DFO), dithiothreitol (DTT), and cycloheximide (CHX) were purchased from Sigma Aldrich (St. Louis, MO, USA). M-MLV reverse transcriptase was purchased from Promega (Madison, WI, USA). Western blot detection kit (Absignal) was purchased from Abclon (Seoul, South Korea). Control and PIN1 targeting si-RNA were purchased from Santa Cruz Biotechnology (Santa Cruz, CA, USA). All other chemicals used were in the purest form available commercially.

### Cell culture

Human colon cancer (HCT116) and human embryonal kidney (HEK293T) cell lines obtained from American type culture collection were maintained in DMEM containing 10% FBS at 37°C in a 5% CO_2_ / 95% air incubator. When necessary, cells were exposed to hypoxia (1% O_2_) by incubation at 37°C in a 5% CO_2_ / 94% N_2_ hypoxia chamber (Forma Scientific, Marietta, OH, USA).

### Lentiviral production and infection

Lentiviruses were produced by transfecting HEK293T cells using lentiviral vectors [20]. In brief, PIN1 shRNA lentiviral vector was transfected with VSV-G-, pLP1- and pLP2-expressing plasmids into HEK293T cells, and lentiviral supernatants were collected at 48 h and 72 h post-transfection. HCT116/5xHRE-ODD-luc cells were infected with PIN1-shRNA or control virus with 5 μg/ml polybrene, and stable clones were selected using 1 μg/ml puromycin, as described previously [21]. To overexpress 5xHRE-ODD-luc and knockdown PIN1 expression, cells were sequentially infected with 5xHRE-ODD-luc retrovirus, and a day later with control or PIN1-shRNA lentiviruses, followed by selection of stable cells.

### Western blot analysis

Sodium dodecyl sulfate-polyacrylamide gel electrophoresis (SDS-PAGE) was performed on cell lysates using 8–12% acrylamide gels according to the method as described by previously [22]. Proteins transferred to polyvinylidene difluoride (PVDF) membranes (Millipore, Billerica, MA, USA) were blocked overnight at 4°C in Tris-buffered saline containing 0.1% Tween 20 (TBS/T) and 5% skim milk powder. The membranes were then incubated with a 1:1000 dilution of HIF-1α, PIN1, VEGF-A and actin antibodies. Blots were washed with TBS/T, and then incubated with a 1:5000 dilution of horseradish peroxidase-conjugated anti-rabbit or anti-mouse IgG antibody. Proteins were detected using Western blotting detection kit. The intensities of bands were quantified using a gelpro32 for Window Program.

### Immunoprecipitation

Cells treated under hypoxic and normoxic conditions for appropriate time were collected for immunoprecipitation. Briefly, cells were lysed with NET-NL lysis buffer [50 mM Tris-HCl (pH 7.5), 5 mM EDTA, 150 mM NaCl, 1 mM DTT, 0.5% NP-40, 0.2 mM PMSF, and one tablet of Protease Inhibitor Cocktail (Roche, Basel, Switzerland)] for co-immunoprecipitation. Following centrifugation at 12,000 rpm, the supernatant was precleared with 20 μl protein A agarose beads (Millipore, Billerica, MA, USA) coupled with mouse or rabbit IgG for 1 h and then incubated with the indicated antibodies overnight at 4°C. The cells were exposed to 20 μl protein A agarose beads for 4 h. The beads were washed 3 times with 1 ml NET-NL lysis buffer. The precipitates were dissolved in the SDS loading buffer for Western blotting analysis.

### Isothermal titration calorimetry (ITC)

ITC experiments were performed in a Microcal iTC200 (Malvern, Worcestershire, UK). PiB (10 mM) was titrated into 1 mM GST-PIN1 in 5 μl increments in PBS at 25°C. Data were fitted to an N identical binding sites model with Microcal analysis launcher Software.

### Immunocytochemistry

Cells were plated on the chamber slide and grown to 70% confluence in complete growth media. PIN1 siRNA was transfected into cells with lipofectamine RNAi-MAX reagents (Invitrogen, Waltham, CA, USA) according to the manufacturer’s instructions. After 72 h transfection, cells were exposed to 1% O_2_ in a hypoxia chamber for additional 4 h. After fixation with 4% paraformaldehyde solution for 15 min at room temperature (RT), samples were incubated with blocking agents (0.1% Triton in PBS containing 10% bovine serum albumin), washed with PBS and co-incubated with HIF-1α (1:200) and PIN1 (1:100) primary antibodies for 1 h at RT. After washing with PBS, samples were co-incubated with diluted (1:5000) FITC-conjugated anti-rabbit and anti-mouse IgG secondary antibodies for additional 1 h at RT. The cells were then examined under a fluorescence microscope (Nikon, Tokyo, Japan) or a confocal microscope (Leica, Berlin, Germany).

### *In vitro* glutathione *S*-transferase (GST) pull-down assay

HCT116 cells were transiently transfected HA-tagged HIF-1α. GST fusion PIN1 proteins were collected on glutathione-Sepharose beads (Amersham, Buckinghamshire, UK) and incubated overnight at 4°C with 1 mg of cell lysate. The bound proteins were denatured in sample buffer and separated by 8 or 12% SDS-PAGE, and expression was detected by Western blot analysis. To determine whether catalytic inactivation of PIN1 could inhibit its interaction with HIF-1α, PiB was reacted with GST-PIN1, and then the complex was reacted with cell lysate transfected with HA-tagged HIF-1α. For dephosphorylation of HA- HIF-1α, endogenous HA-HIF-1α was purified by use of the HA tagged protein purification kit (MBL, JP) according to manufacturer’s instructions. The HA-HIF-1α was incubated with or without lamda phosphatase (NEB, MA, USA) for 1 h at 30°C in the phosphatase buffer supplied by the manufacturer.

### *In situ* proximity ligation assay (PLA)

PLA was carried out using the DUOLink^TM^ kit (OLINK, Uppsala, Sweden) according to manufacturer’s instructions. In brief, HCT116 cells on glass coverslips were fixed, permeabilized, and blocked with blocking solution (0.1% Triton in PBS containing 5% bovine serum albumin) and incubated with the antibodies against HIF-1α (1:20) and PIN1 (1:10) for 1 h at 37°C. PLA plus and minus affinity probes were then added and incubated for additional 1 h at 37°C. The probes were hybridized using a ligase to be a closed circle. The DNA was then amplified (a rolling-circle amplification) and detected by fluorescence microscopy.

### Protein stability assay

The HCT116 cells after 72 h transfection with PIN1 si-RNA were preincubated under hypoxic conditions for 4 h. Then, the cells were treated with 10 μM cycloheximide under hypoxic conditions to block protein synthesis. The cells were collected for Western blotting analysis.

### Limited chymotrypsin digestion assay

The purified HA-HIF-1α derived from parent cells untreated or treated with GFP-PIN1 was subjected to digestion with 50 ng chymotrypsin (SERVA, Heidelberg, Germany), and incubated at 37°C for the indicated time. Digestion was terminated by the addition of SDS-PAGE loading buffer and boiling of the samples. Processing of the HA-HIF-1α was analyzed using 8% SDS-PAGE.

### Reverse transcription-PCR analysis

Cells were lysed with TRIzol^®^, and total RNA was isolated with chloroform and isopropyl alcohol. RNA was subjected to reverse transcription M-MLV reverse transcriptase according to the manufacturer’s instructions. The cDNA was amplified via 35-cycle PCR with DNA polymerase and primers for HIF-1α (sense, 5’-CAAGACTTTCCTCAGTCGACA-3’; antisense, 5’-GGGAGAAAATCAAGTCGTG-3’), PIN1 (sense, 5’-AGCAGCAGTGGTGGCAAAAA-3’; antisense, 5’-GGCCAGAGACTCAAAGTCCT-3’), and VEGF-A (sense, 5’-AGTGGTGAAGTTCATGGATGTC-3’; antisense, 5’-TGCTCTATCTTTCTTTGGTCTG-3’). The PCR products were analyzed with 1.2% agarose gel and stained with SYBR^®^Green for visualization.

### Luciferase reporter gene assay

Cells were subcultured in 12-well plates at a density 5 x 10^4^ cells/well one day before transfection. To investigate the effect of PIN1 silencing on EPO-HRE reporter activity, cells were transfected with PIN1 siRNA or mock siRNA for 72 h at 37°C. The cells were transfected with plasmids including luciferase-linked reporter gene (EPO-HRE luc) and β-galactosidase (β-gal) expression vector. After 24 h transfection, cells were lysed, and luciferase activities were measured. The β-gal activity was used to normalize transfection efficiency.

Bioluminescence image was obtained using a luciferase assay kit (Applied Biosystems, Carlsbad, CA, USA). The plates were washed twice with PBS, and then lysis solution was added to each well. Cell lysates were transferred to a 96 well microplate and luciferase activities were measured by a Wallac 1420 VICTOR3 V (PerkinElmer Life and Analytical Sciences, Shelton, CT, USA) luminometer.

### Tube formation assay

Tube formation assays were conducted to examine the effect of PIN1 silencing on the *in vitro* angiogenesis in human umbilical vein endothelial cells (HUVEC). Briefly, a 96-well plate was coated with 55 μl of Matrigel, which was allowed to solidify at 37°C for 1 h. HUVEC cells (2 x 10^4^ per well) were planted into Matrigel-coated 96-well plates and cultured in the Vasculife VEGF Medium Complete Kit (Lifeline cell technology, Frederick, MD, USA) with 10% FBS under both hypoxic and normoxic conditions for the indicated times. The tube-like networks were visualized under a microscope.

### *In vitro* hypoxia imaging with the IVIS bioluminescence system

For establishing of stable cell line, human colon cancer (HCT116) cells were transfected with a HIF-1-dependent reporter gene (5xHRE-ODD-luc). The cells were exposed to hypoxia (1% O_2_) in a hypoxic chamber. In normoxic conditions, ODD-luc protein was degraded because of the presence of oxygen-dependent degradation domain (ODD). However, in hypoxic conditions, the expression of ODD-luciferase fusion protein was enhanced by 5 copies of hypoxia-response element (5xHRE). Hypoxia was also induced by the hypoxia-mimetic agent, DFO. HCT116/5xHRE-ODD-luc cells (1 × 10^5^) were seeded in 24 well plates. After 24 h, the cells were treated with DFO (400 μM) and/or PiB (20 μM) for additional 8 h. To measure the effect of ATRA on HIF-1 reporter luciferase activity, HCT116/5xHRE-ODD-luc cells were seeded in the 96 well plate. After 24 h, the cells were treated with ATRA with indicated concentrations for 48 h. The cells were subjected to hypoxia (1%) for additional 24 h. To assess the effect of silencing of PIN1 on transcriptional activity of HIF-1, HCT116/5xHRE-ODD-luc cells infected with PIN1-shRNA or control viruses were seeded in 96 well plates. After 24 h, the cells were cultured under hypoxic conditions as above. To obtain bioluminescence images, 100 μl of D-luciferin (0.3 μg/μl) was treated to each well. Bioluminescent images were acquired using the IVIS Lumina imaging system (Xenogen Corp., Alameda, CA, USA). The images were analyzed using LIVINGIMAGE V. 2.50.1 software (Xenogen Corp., Alameda, CA, USA).

### *In vivo* bioluminescence imaging in the colon cancer xenograft model

Animal experiments were conducted in accordance with the Guide for the Care and Use of Laboratory Animals and approved by the Institutional Animal Care and Use Committee of Seoul National University (SNU protocol #140224–4). Six-week-old male BALB/c nu/nu mice weighing 20 g on average were kept under specific-pathogen-free conditions. Mice were acclimated to the environment for 1 week before the experiment. HCT116/5xHRE-ODD-luc cells were pretreated with PiB (20 μM), and the cells (1 x 10^6^ each) were subcutaneously transplanted in the each thigh of mice to make xenografts. Mice were monitored every 4 day from 0 to 16 day after tumor inoculation. All mice were checked for their body condition and health by use of specific criteria, such as weight loss, weakness/inability to obtain feed or water, inappetence, moribund state, and infection. Data on body weight changes are provided in **S1 Table**. The tumor size was measured using calipers at 16 days later. Tumor volumes were calculated according to the following formula: (length) x (width/2)^2^ x π. Bioluminescence imaging was obtained by the IVIS Luminar (Xenogen Corp., Alameda, CA, USA). A region of interest (ROI) was drawn over the tumor margins. For subcutaneous tumors, the maximum allowable size was 20 mm in diameter according to our institutional guidelines. In our study, the mean tumor size was 7.74 mm in diameter, and the maximum tumor size was 17.91 mm in diameter. The raw data regarding the size, the width and the volume of tumors over time are provided in **S2 Table**. The animals were sacrificed in a CO_2_ chamber which is an acceptable method for euthanasia of animals.

### *In vivo* Matrigel plug assay

To assess angiogenesis *in vivo*, HCT116 cells were re-suspended in serum-free medium at 1.5 x 10^7^ cells/ml, and aliquots of the cells (0.2 mL) were mixed with 0.4 mL of growth-factor-reduced Matrigel in the absence or presence of PiB. The mixtures were injected subcutaneously into both sites of flank of male BALB/c nu/nu mice. The mice were euthanized 10 days after the implantation. The Matrigel plugs were surgically removed from the mice. For macroscopic analysis of angiogenesis, hemoglobin (Hb) content in Matrigel was quantified by using Drabkin’s reagent which was added to homogenized Matrigel. After through mixing, absorbance at 540 nm was measured to estimate the Hb content. In another experiment, HCT116/5xHRE-ODD-luc cells were infected with PIN1-shRNA or control viruses. The cells were re-suspended in 10% DMEM medium at 1.5 x 10^7^ cells/ml, and aliquots of the cells (0.1 mL) were mixed with 0.1 mL of growth factor-reduced Matrigel. The mixtures were injected subcutaneously into both sites of male BALB/c nu/nu mice. Mice were monitored every 2 day for 2 weeks after tumor inoculation. Bioluminescence imaging was obtained by the IVIS Luminar. The tumor size was measured using calipers at 14 days later. The mice were then euthanized, and Hb contents in the Matrigel plugs were measured by the manufacturer.

### Immunohistochemistry and Immunofluorescence

Immunohistochemistry was performed to confirm the expression of luciferase, VEGF, CD31, HIF-1α, and PIN1. Tumor tissues were fixed in 3.7% paraformaldehyde for 24 h. Paraffin embedded tissues were serially sectioned in 4 μm, and mounted on a slide. For antigen retrieval, citrate buffer (DakoCytomation, Glostrup, Denmark) was used. The staining conditions for primary antibodies were as follows; anti-CD31 (1:200), anti-luciferase (1:200), anti-VEGF (1:200), anti-HIF-1α (1:200), and anti-PIN1 (1:200). The secondary antibodies were treated at RT for 1 h; biotinylated anti-rabbit (1:500), biotinylated anti-goat (1:500), anti-rabbit Alexa488 (1:200) and anti-mouse Alexa568 (1:200). The tissue sections treated with anti-CD31, anti-luciferase, and anti-VEGF were amplified with complex of avidin-biotin peroxidase, and developed using DAB.

### Statistical Analysis

The results were presented as means ± SD. To determine the statistical significances, the student's unpaired *t*-test was used, and P-values less than 0.05 were considered significant.

## Results

### PIN1 physically interacts with HIF-1α

**Fig 1:**
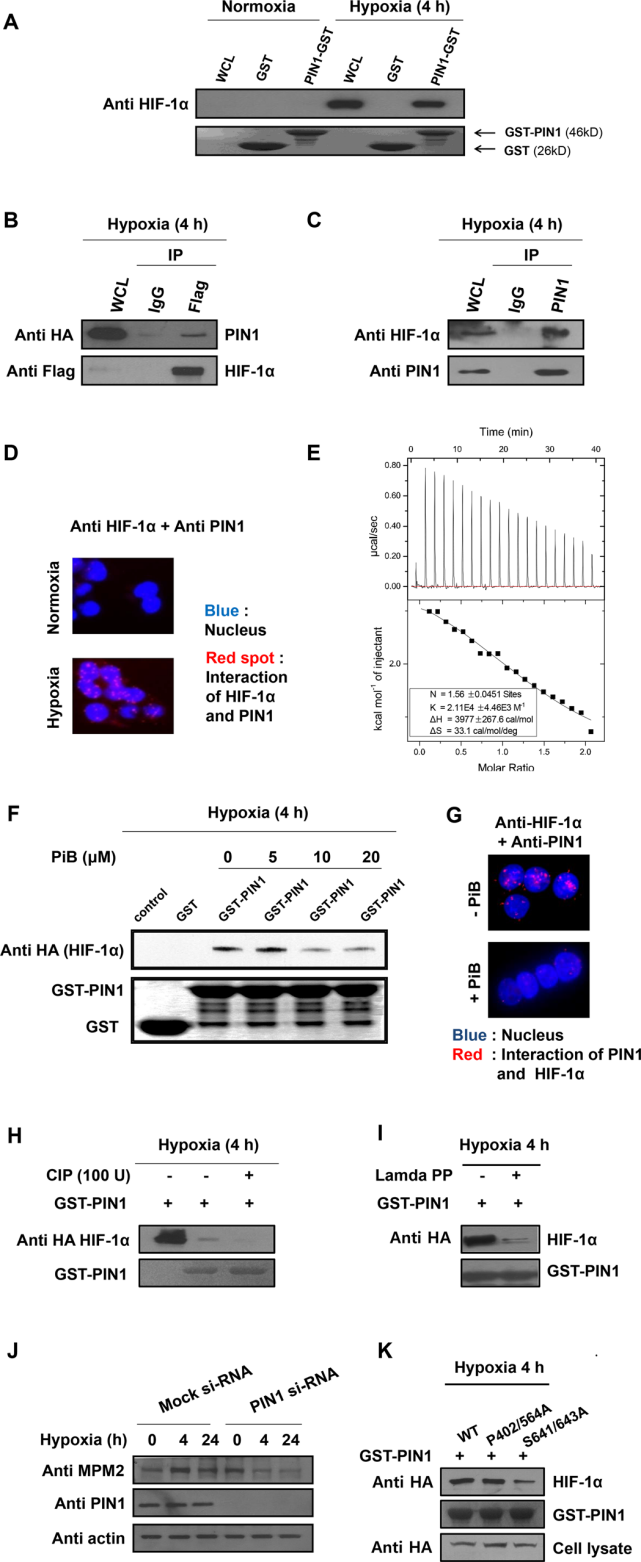
PIN1 physically interacts with HIF-1α in a phosphorylation-dependent manner. A) *In vitro* GST pull down assay. The cell lysates were incubated with GST or GST-PIN1 fusion protein, followed by addition of the GST beads. The precipitates were immunoblotted with anti-HIF-1α to show the bound HIF-1α and reprobed with anti-GST to show the precipitated GST and GST-PIN1. B) Interaction of HIF-1α and PIN1 in HCT116 cells. Cells were transfected with Flag-tagged HIF-1α and HA-PIN1, and stimulated with hypoxia for 4 h. The cell lysates were immunoprecipitated with anti-Flag antibody, and the precipitates were fractionated by SDS-polyacrylamide gel electrophoresis and blotted with anti-HA antibody. C) Association of endogenous PIN1 and HIF-1α. HCT116 cells were subjected to hypoxia for 4 h. Cell lysates were incubated with either normal IgG or anti-PIN1 as labeled and blotted with anti HIF-1α. D) Binding of HIF-1α and PIN1 *in situ*. The HCT116 cells were incubated under normoxia or hypoxia. Interaction of HIF-1α and PIN1 was visualized by Duolink analysis. HIF-1α and PIN1 were co-labeled with antibodies. Nuclei were counter stained with DAPI (blue). Scale bar, 20 μm. E) ITC indicates that PiB binds PIN1. Heat evolution as a function of adding increasing amounts of PiB to GST-PIN1. The heats of dilution were measured separately, and found to be <1% of the signal at the start of titration. Fitting the ITC data with Microcal analysis launcher software indicates that PiB binds to GST-PIN1. Values shown on the figure are from the fit of the displayed dataset. K = 2.11E4 ± 4.46E3M^-1^, N = 1.56 ± 0.0451 sites, ΔH = 3.977 ± 0.2676 Kcal/mol, ΔS = 33.1 cal/mol/deg. F) *In vitro* GST pull down assay. The cell lysates were incubated with GST or GST-PIN1 fusion protein, followed by treating with PiB and addition of the GST beads. The precipitates were immunoblotted with anti-HIF-1α to show the bound HIF-1α and reprobed with anti-GST to show the precipitated GST and GST-PIN1. G) Binding of HIF-1α and inactivated PIN1 *in situ*. HCT116 cells were incubated with or without PiB (20 μM). Interaction of HIF-1α and inactivated PIN1 was visualized by Duolink analysis. HIF-1α and PIN1 were co-labeled with antibodies. Nuclei were counter stained with DAPI (blue). H) CIP (50 U) was added to the supernatants at 30°C for the indicated time periods. Following incubation, GST or GST-PIN1 proteins were incubated with the supernatants for 4 h and then were pulled down with GST beads. Following incubation, reactions were stopped by the addition of SDS sample buffer, followed by SDS-PAGE. I) HA-HIF-1α proteins were purified using a commercially available kit. The proteins were treated with or without lamda phosphatase for 1 h at 30°C. Then GST-PIN1 proteins were incubated with purified HA-HIF-1α proteins and were pulled down with GST beads. The proteins were resolved in SDS-polyacrylamide gels and detected by immunoblotting. J) Cells were transfected with scrambled siRNA as a negative control or PIN1-siRNA for 72 h and treated with hypoxia for 4 h. Cell lysates were incubated with either normal IgG or anti-MPM2 as labeled and blotted with anti-PIN1. K) HA-HIF-1α, HA-HIF-1α^S402/564A^, and HA- HIF-1α^S641/643A^ were transfected in HCT116 cells. Whole cell extracts of HCT116 cells were prepared for pull down assays with GST-PIN1 proteins. The pull downed fractions were s with anti-HA antibody.

Given that HIF-1α contains multiple Ser/Thr-Pro motifs (13 pSer-Pro and 2 pThr-Pro), HIF-1α could be a putative substrate of PIN1. To test this possibility, the GST pull-down assay was conducted using a whole cell lysate and GST-conjugated- or GST-PIN1-conjugated Sepharose 4B beads. GST-PIN1 fusion protein, but not GST protein, pulled down a substantial amount of HIF-1α from lysates of HCT116 cells subjected to hypoxia, indicative of a direct interaction between PIN1 and HIF-1α (**Fig 1A**). In another experiment, HEK293T cells were co-transfected with expression vectors encoding Flag epitope-tagged HIF-1α and HA-tagged PIN1, and whole-cell lysates were immunoprecipitated with anti-Flag antibody. As shown in **Fig 1B**, HIF-1α bound to exogenous PIN1. To determine whether the interaction of PIN1 with HIF-1α was detectable at the endogenous level, we conducted immunoprecipitation with anti-HIF-1α antibody or anti-PIN1 antibody. **Fig 1C** illustrates endogenous PIN1 interacting physically with HIF-1α. To further verify the potential interaction between HIF-1α and PIN1, we performed a Duolink *in situ* proximity assay, in which the direct interaction of the two proteins can be visualized by fluorescence staining. We found that fluorescent spots reflecting interaction between HIF-1α and PIN1 localized predominantly in nucleus under hypoxic conditions, whereas such interaction did not occur under normoxic conditions (**Fig 1D**).

In order to further verify the interaction between PIN1 and HIF-1α, we utilized PiB that has been shown to inhibit the catalytic activity of PIN1 by directly binding to the enzyme [23]. We confirmed that PiB did bind to PIN1 as assessed by the ITC assay (**Fig 1E**). As illustrated in **Fig 1F**, the binding of PIN1 to exogenous HIF-1α was dramatically reduced after the PiB treatment. The interaction between endogenous HIF-1α and PIN1, as determined by the *in situ* proximity assay, was also attenuated in the presence of PiB (**Fig 1G**).

### Phosphorylation of HIF-1α is necessary for its interaction with PIN1

PIN1 interacts with its substrates in a phosphorylation-dependent manner. To determine whether the interaction between PIN1 and HIF-1α is also mediated through phosphorylation, we pretreated the cell lysates with the calf intestinal alkaline phosphatase (CIP) prior to the GST pull-down assay. Treatment of cell lysates with CIP reduced the interaction between PIN1 and HIF-1α, suggesting a phosphorylation-dependent interaction between two proteins (**Fig 1H**). In addition, a nonspecific protein phosphatase, lamda phosphatase, strongly abrogated the interaction of PIN1 and purified HA-HIF-1α (**Fig 1I**). To determine whether HIF-1α was phosphorylated at Ser/Thr-Pro motifs, we employed the monoclonal antibody MPM2, capable of specifically recognizing the pSer/Thr-Pro motifs. When the expression of PIN1 was silenced by RNAi, the protein levels of MPM2 were also reduced (**Fig 1J**). ERK phosphorylates HIF-1α at Ser^641^ and Ser^643^ residues [8] both of which constitute PIN1 consensus motifs (pSer-Pro). The phosphorylation of HIF-1α at these residues by ERK1/2 was found to be essential for its interaction with PIN1 [24]. In agreement with these findings, we also noticed that substitution of Ser^641^ and Ser^643^ for alanine markedly diminished the levels of the HIF-1α and PIN1 complex (**Fig 1K**). HIF-1α undergoes degradation through proline-hydroxylation at both pVHL binding sites (Pro402 and Pro564). However, mutation of both proline residues did not influence the interaction between HIF-1α and PIN1 (**Fig 1K**). Taken together, these results indicate that PIN1 interacts specifically with HIF-1α phosphorylated at the Ser^641^ and Ser^643^ in hypoxic conditions.

### PIN1 silencing leads to reduction in the protein, but not mRNA, levels of HIF-1α

**Fig 2:**
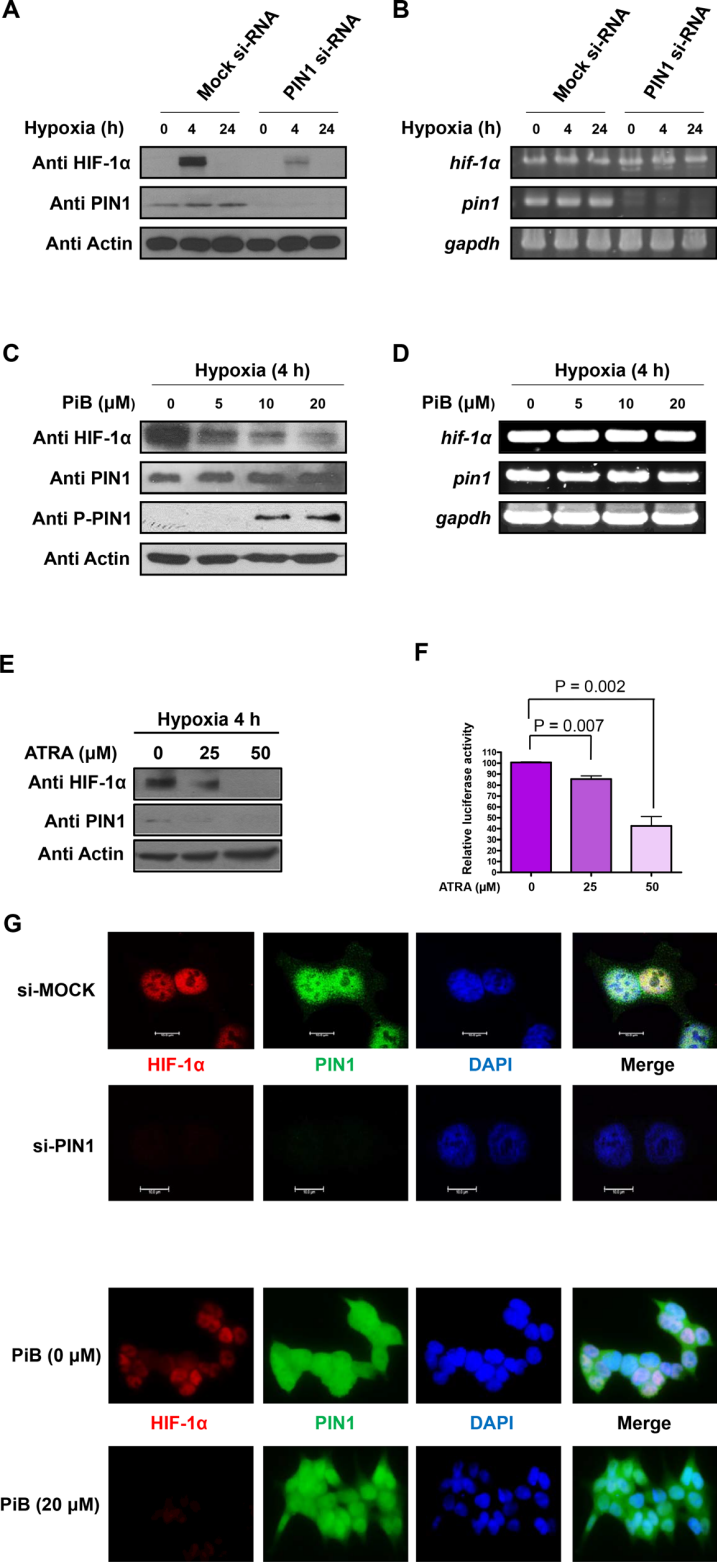
PIN1 silencing leads to decreases in the protein levels of HIF-1α. A) Silencing of PIN1 expression via RNAi reduces the protein levels of HIF-1α in HCT116 cells. Western blot analysis was performed with 50 μg total cell lysate from HCT116 cells transfected with control or PIN1 si-RNA. B) mRNA levels of HIF-1α and PIN1 were determined by RT-PCR analysis at the indicated time points under hypoxic conditions. GAPDH was used as internal controls. C) The PIN1 inhibitor, PiB, reduces the protein levels of HIF-1α in HCT116 cells. HCT116 cells were mock-treated or treated with PiB and harvested 8 h after treatment for the analysis of the HIF-1α and PIN1 levels. D) HCT116 cells were mock-treated or treated with PiB and harvested 8 h later. mRNA levels of HIF-1α and PIN1 were determined by RT-PCR analysis at the indicated time points under hypoxic conditions. GAPDH was used as internal controls. E) ATRA decreases the expression of HIF-1α and PIN1 in HCT116/5xHRE-ODD-luc cells in dose-dependent manner. F) The luciferase activity of HRE in HCT116/5xHRE-ODD-luc cells treated ATRA was reduced in dose-dependent manner. G) Nuclear co-localization of HIF-1α and PIN1 was determined by immunocytochemistry. HCT116 cells plated on the chamber slide were transfected PIN1 si-RNA or treated PiB and grown at 1% O_2_ in a hypoxia chamber for additional 4 h. The samples were co-incubated with anti HIF-1α and PIN1 antibodies and then co-incubated with FITC-conjugated secondary antibodies. Lastly, samples were stained with DAPI. The signals were detected using a confocal microscope or a fluorescent microscope. Scale bar, 10 μm.

To confirm that the stability of HIF-1α is regulated by PIN1 in hypoxic conditions, expression of PIN1 was silenced by RNAi. When PIN1 was knockdown in HCT116 cells, the protein levels of HIF-1α were markedly reduced (**Fig 2A**). In contrast, the mRNA levels of HIF-1α were not affected by PIN1 depletion (**Fig 2B**). These results suggest that PIN1 regulates the expression of HIF-1α at the post-translational level in HCT116 cells. Molecular modeling analysis demonstrates that PiB binds PIN1 at the catalytically active site and thereby inhibits PPIase activity of PIN1 in a competitive manner by masking its substrate binding sites [23]. Consistent with the finding from silencing of PIN1, pharmacologic inhibition of its activity with PiB also significantly reduced the protein levels of HIF-1α in HCT116 cells (**Fig 2C**). Again, the mRNA levels of HIF-1α were constant under the same experimental conditions (**Fig 2D**). When HCT116 cells were treated with ATRA, a selective PIN1 inhibitor, the expression level of HIF-1α protein was similarly decreased in dose-dependent manner (**Fig 2E**). Moreover, the HIF-1α transcriptional activity was suppressed in HCT116/5xHRE-ODD-luc cells treated with ATRA (**Fig 2F**). These findings suggest that the isomerase activity of PIN1 is important for stabilization of HIF-1α. The subcellular co-localization of HIF-1α and PIN1 was verified by immunofluorescence microscopy using anti-HIF-1α and anti-PIN1 antibodies. The distribution of HIF-1α and PIN1 was predominantly nuclear under hypoxic conditions. The nuclear accumulation of HIF-1α was attenuated by either silencing of PIN1 (**Fig 2G**, left panel) or PiB treatment (**Fig 2G**, right panel).

### PIN1 prolongs the stability of HIF-1α possibly by inducing a conformational change

**Fig 3:**
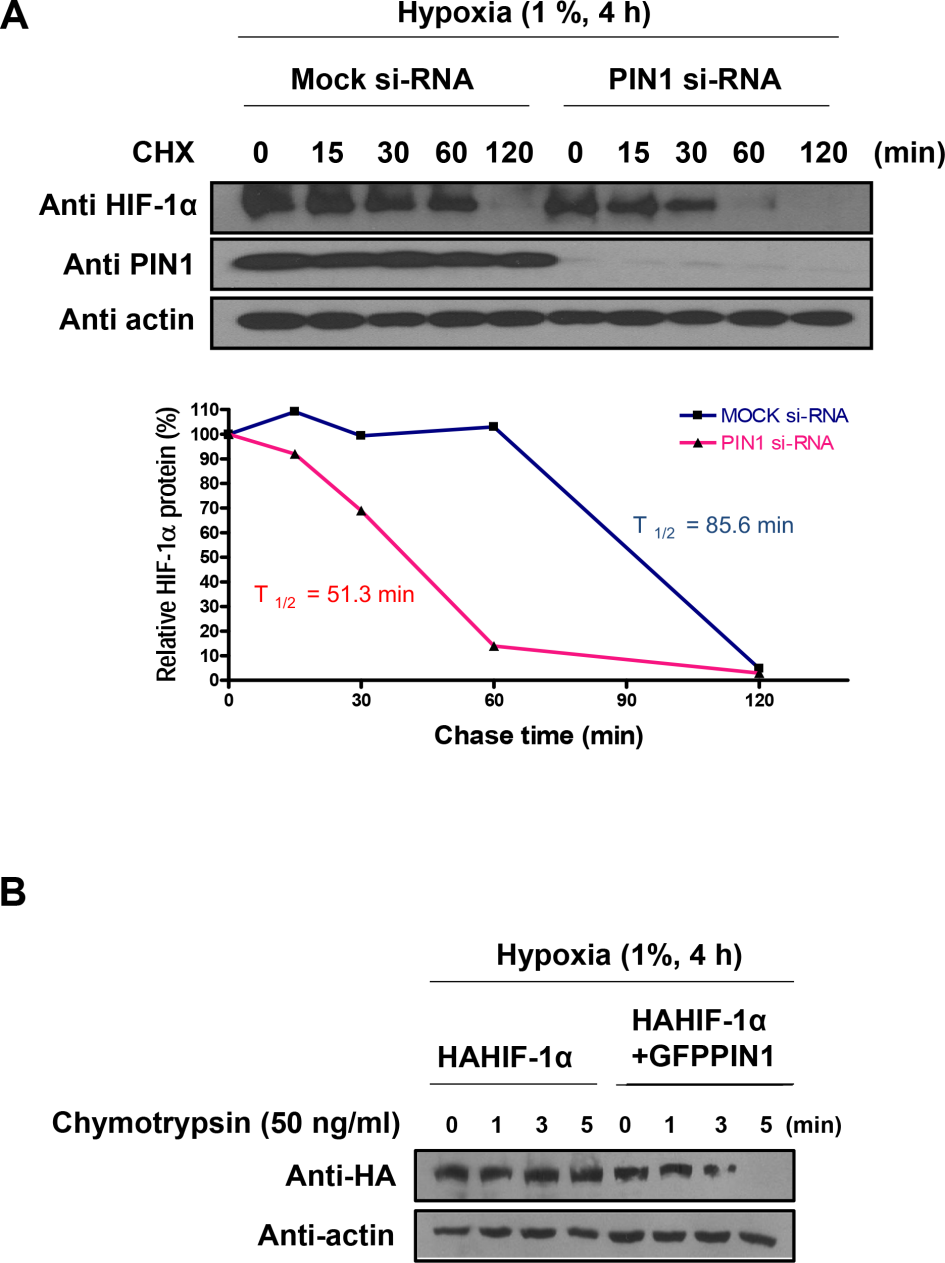
PIN1 enhances the stability of HIF-1α. A) Lysates of cells were transfected with DNA-based siRNA expression vectors for 72 h, then exposed to hypoxia for 4 h, and then treated with CHX (10 μM) for the indicated time periods under hypoxic condition were used for Western blotting analysis with anti-HIF-1α and anti-PIN1. Relative amounts of the HIF-1α signals, normalized to the values obtained in the absence of CHX, were plotted and used to calculate half-lives. Relative levels of the HIF-1α signals were plotted and used to calculate half-lives. PIN1 depletion significantly reduced the stability of HIF-1α half-life. B) PIN1 induces the conformational change of HIF-1α. Limited proteolysis of purified HA-HIF-1α derived from HCT116 cells transfected GFP-PIN1 or GFP. The purified HA-HIF-1α was subjected to partial chymotrypsin digestion and analyzed by Western blotting.

To more precisely assess whether enhanced PIN1 level in hypoxic cells is associated with increased HIF-1α stability, we measured the HIF-1α half-life (**Fig 3A**). For this purpose, endogenous PIN1 in human colon cancer (HCT116) cells was knocked down using PIN1 si-RNA, and stability of endogenous HIF-1α protein was monitored by a cycloheximide chase experiment. Silencing of endogenous PIN1 resulted in a rapid and significant decrease in the stability of HIF-1α under hypoxic conditions (si-mock t_1/2_ = 85.6 min vs. si-PIN1 t_1/2_ = 51.3 min). To determine whether the conformational change of HIF-1α could affect stabilization by altering its interaction with PIN1, we performed a partial proteolytic cleavage assay. The HA-HIF-1α purified from HCT116 cells transfected with the GFP-PIN1 was more susceptible to limited chymotrypsin digestion, as opposed to the HA-HIF-1α derived from HCT116 cells transfected GFP alone. This finding suggests that HA-HIF-1α may undergo a structural reorganization when bounds to PIN1 (**Fig 3B**).

### PIN1 regulates the hypoxia-induced transcriptional activity of HIF-1

**Fig 4:**
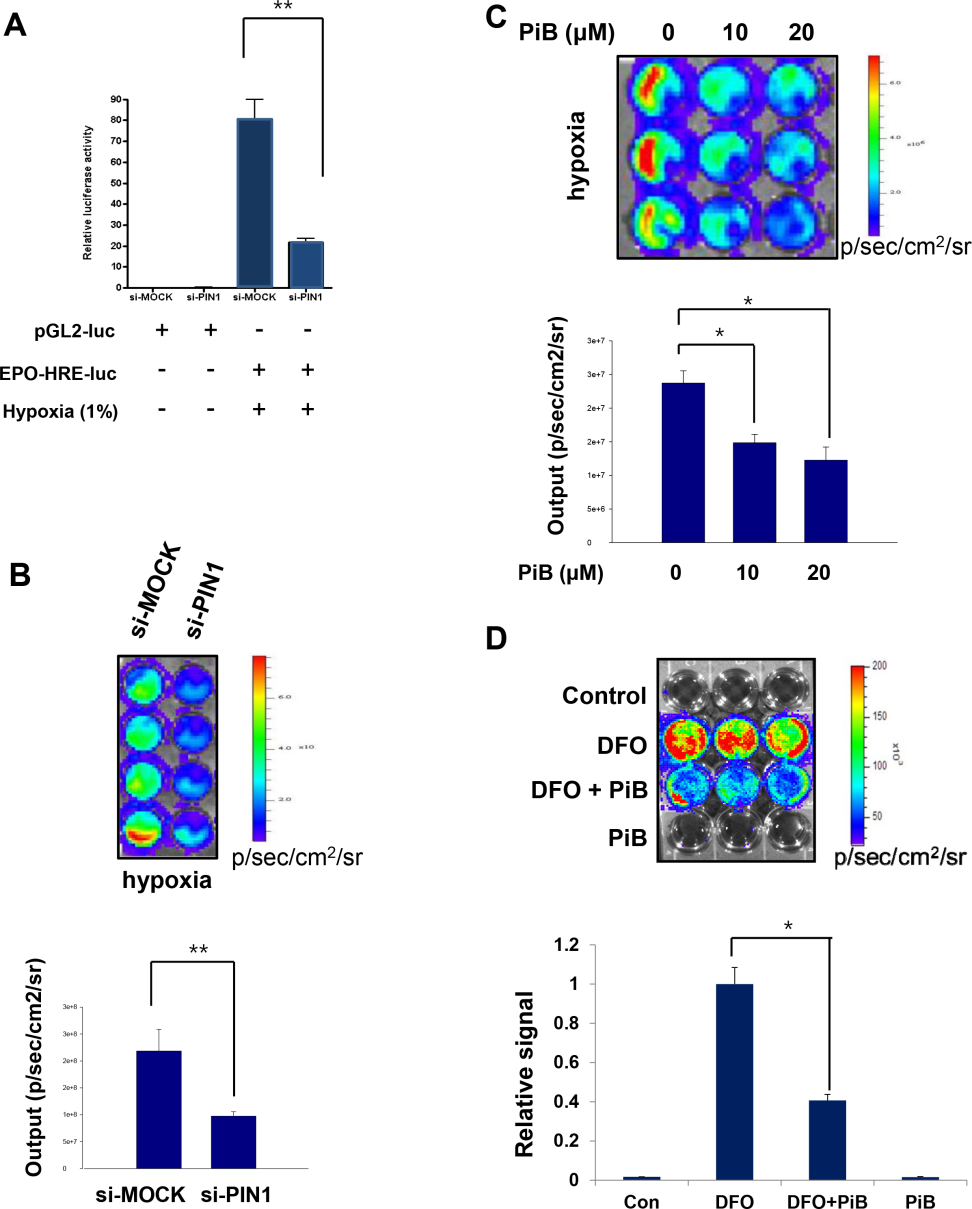
PIN1 regulates the hypoxia induced transcriptional activity of HIF-1. A) Effect of PIN1 knockdown on EPO-HRE-luciferase reporter activity under normoxia or hypoxia for 24 h. HCT116 cells were transfected with control or PIN1 si-RNA for 72 h and then were transfected with pGL2-luc and EPO-HRE-luc for another 24 h. Cells were lysed to analyze luciferase activities, which were normalized against β-galactosidase activities. B, C) *In vitro* HIF-1α bioluminescence assay. 3 x 10^5^ HCT116/5xHRE-ODD-luc cells were transfected with control or PIN1 si-RNA for 24 h (B) or treated with PiB (C) and harvested 8 h after treatment. The cells were incubated in each well of a 96-well-dish in a hypoxia chamber (1% O_2_) for 4 h before the medium was removed, washed and replaced with 1 ml PBS. Immediately after 100 μl luciferin was added into each well, ROIs were acquired with an array of exposure times (1, 30, 60, and 180 s). D) PIN1 regulates transcriptional activity of HIF-1 during hypoxia-mimic conditions. Hypoxia was induced by DFO (400 μM). HCT116/5xHRE-luc cells were treated with DFO only, DFO plus PiB (20 μM), or PiB (20 μM) alone for 8 h. ROIs were analysed by bioluminescence imaging from HCT116/5xHRE-luc cells after various treatments.

Hypoxic stress signaling responses are mediated mainly by HIF-1α-driven transcription of various genes, including EPO-HRE. Hypoxia dramatically increased the luciferase activity of EPO-HRE. Reporter activities of EPO-HRE in HCT116 cells were suppressed by PIN1 siRNA transfection (**Fig 4A**). In addition, we have utilized a HIF-1α luciferase reporter gene construct, 5xHRE-ODD-luc, stably transfected into human colon cancer (HCT116) cells (HCT116/5xHRE-ODD-luc). Cells transfected with PIN1 siRNA showed weak bioluminescence compared to control cells treated with MOCK siRNA (**Fig 4B**). Similarly, luciferase signals from cells treated with the PIN1 inhibitor, PiB exhibited significantly weaker bioluminescence under hypoxia (**Fig 4C**). The hypoxia-mimetic agent DFO, when treated for 8 h to HCT116/5xHRE-ODD-luc cells, generated strong bioluminescence, and PiB treatment significantly decreased DFO-induced luciferase signals (**Fig 4D**).

### PIN1 silencing reduces the mRNA and protein levels of VEGF-A

**Fig 5:**
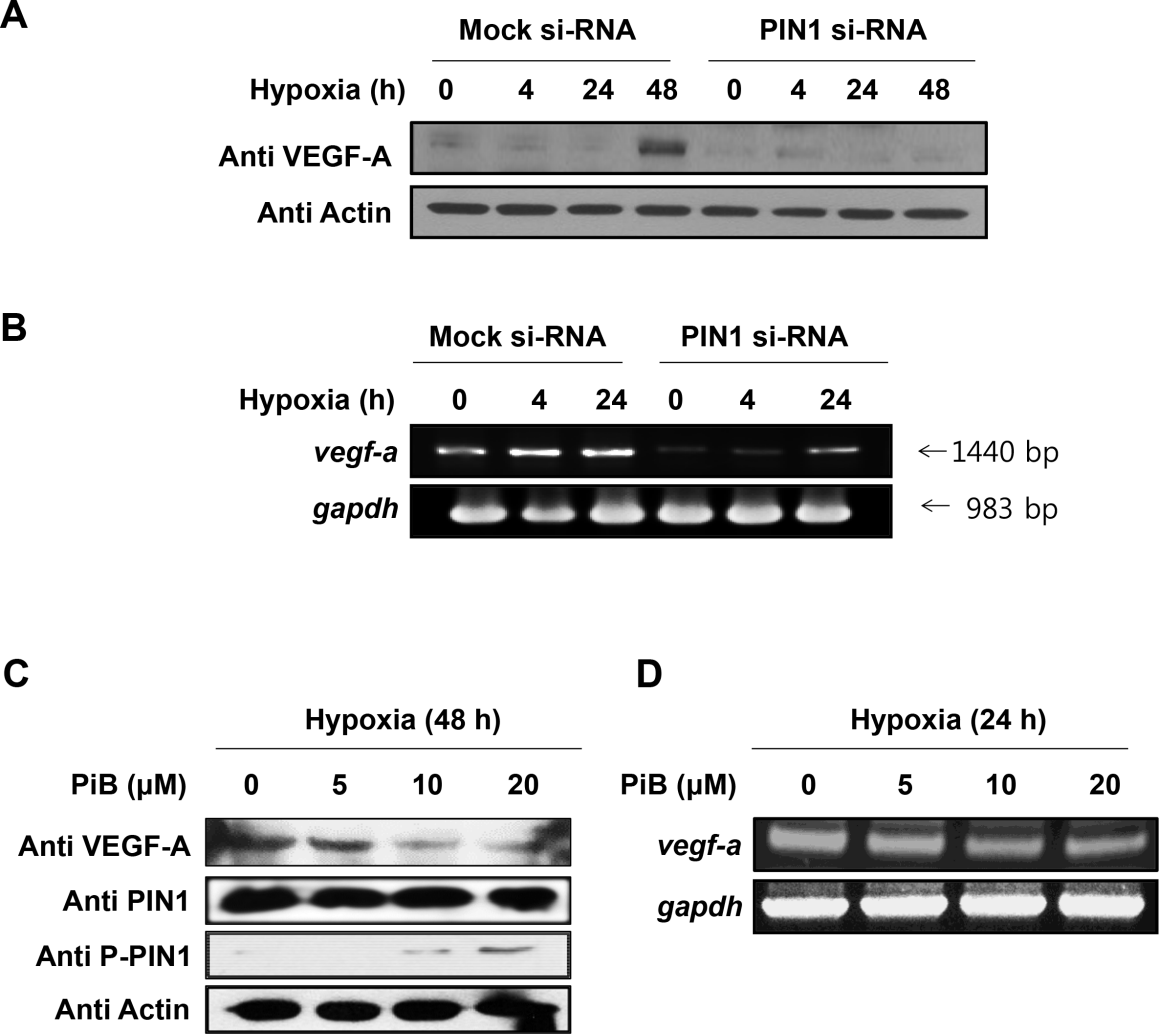
PIN1 silencing reduces the protein levels of VEGF-A. A) The HCT116 cells were transfected with control or PIN1 si-RNA under hypoxia. Western blot analysis was performed with anti-VEGF-A. B) For analysis of VEGF-A mRNA, RT-PCR was performed with 1 μg total RNA prepared from HCT116 cells transfected with control or PIN1 si-RNA under hypoxia. C) The PIN1 inhibitor, PiB, reduces the protein levels of HIF-1α in HCT116 cells. The cells were mock-treated or treated with PiB and harvested 8 h. Western blot analysis was conducted as described **Fig 5A**. D) For the analysis of VEGF-A mRNA, HCT116 cells were mock-treated or treated with PiB and harvested 8 h. RT-PCR analysis was performed as described **Fig 5B**.

VEGF is a potent angiogenesis inducer, and its expression is upregulated by HIF-1. When the PIN1 expression was silenced by siRNA in HCT116 cells, the protein (**Fig 5A**) and mRNA (**Fig 5B**) levels of VEGF-A were reduced. Likewise, hypoxia-induced VEGF-A protein and mRNA expression was attenuated by pharmacologic inhibition of PIN1 (**Fig 5C and 5D**, respectively).

### PIN1 plays a role in enhanced 3D tube formation and *ex vivo* aortic ring vascular sprouting

**Fig 6:**
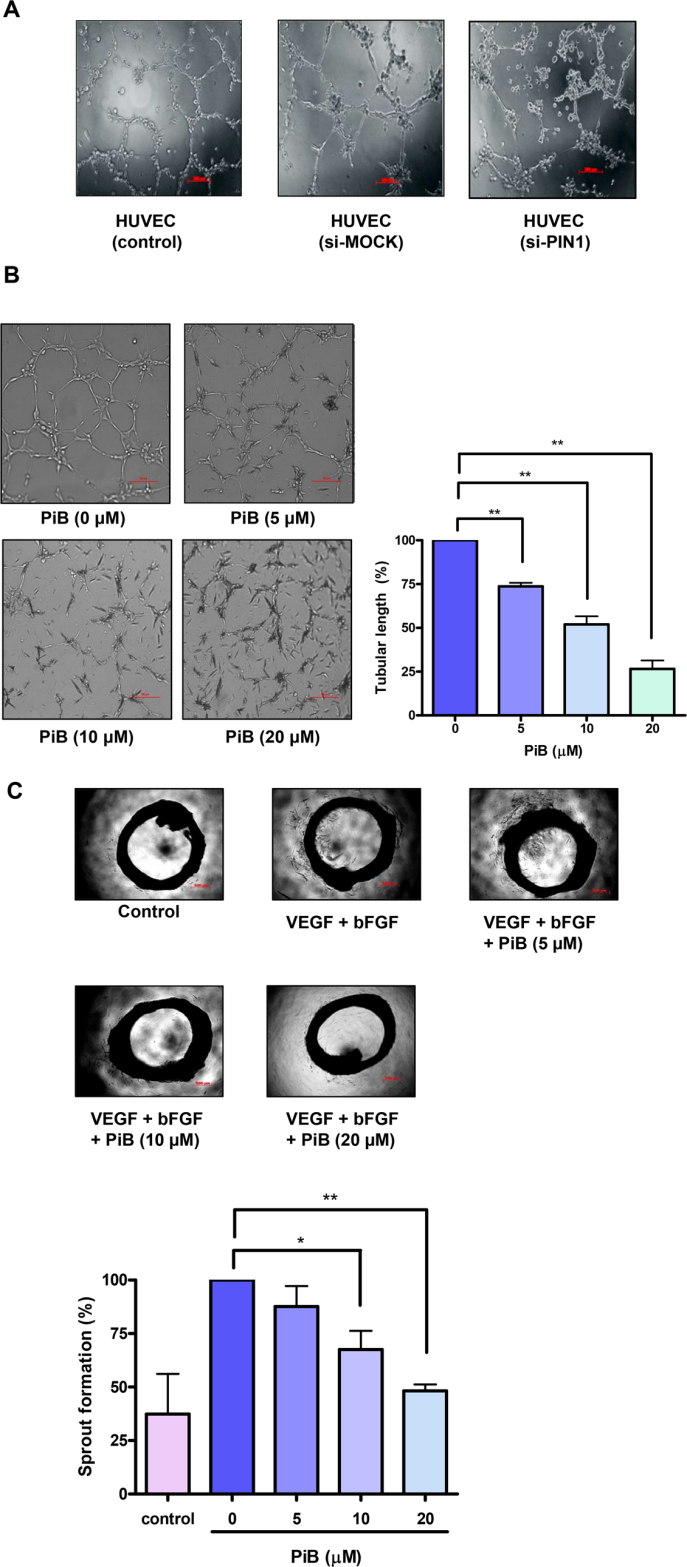
PIN1 enhanced 3D tube formation and *ex vivo* aortic ring vascular sprouting. A) HUVEC were transfected with control or PIN1 si-RNA for 24 h and then were cultured in Matrigel in supplemented media. B) HUVEC were suspended in Matrigel with VEGF in the presence or absence of different concentrations of PiB and maintained for 8 h. Quantification of area of cell alignment was determined by image J software (**P < 0.001). C) Effect of PiB on aortic ring sprouting. Aortic rings were treated with VEGF (100 ng) and bFGF (100 ng) in the presence or absence of different concentrations of PiB for 9 days. Quantification of area of cell alignment was determined by image J software (*P < 0.05, **P < 0.001).

HUVECs plated on Matrigel were cultured overnight in complete media. The formation of endothelial tubules was then quantified. As shown in **Fig 6A**, there was a significant reduction in tube formation in HUVECs transfected with PIN1 siRNA compared with that in control cells and cells transfected with scrambled siRNA. Intact tube structures were developed in control cells, whereas tube formation was significantly disrupted in the cells treated with PiB in a concentration dependent manner (**Fig 6B**). In an aortic ring assay, pretreatment with PiB at indicated doses (1, 5, 10, and 20 μM) also significantly inhibited VEGF induced endothelial cell outgrowth (**Fig 6C**).

### PIN1 enhances tumor angiogenesis *in vivo*

**Fig 7:**
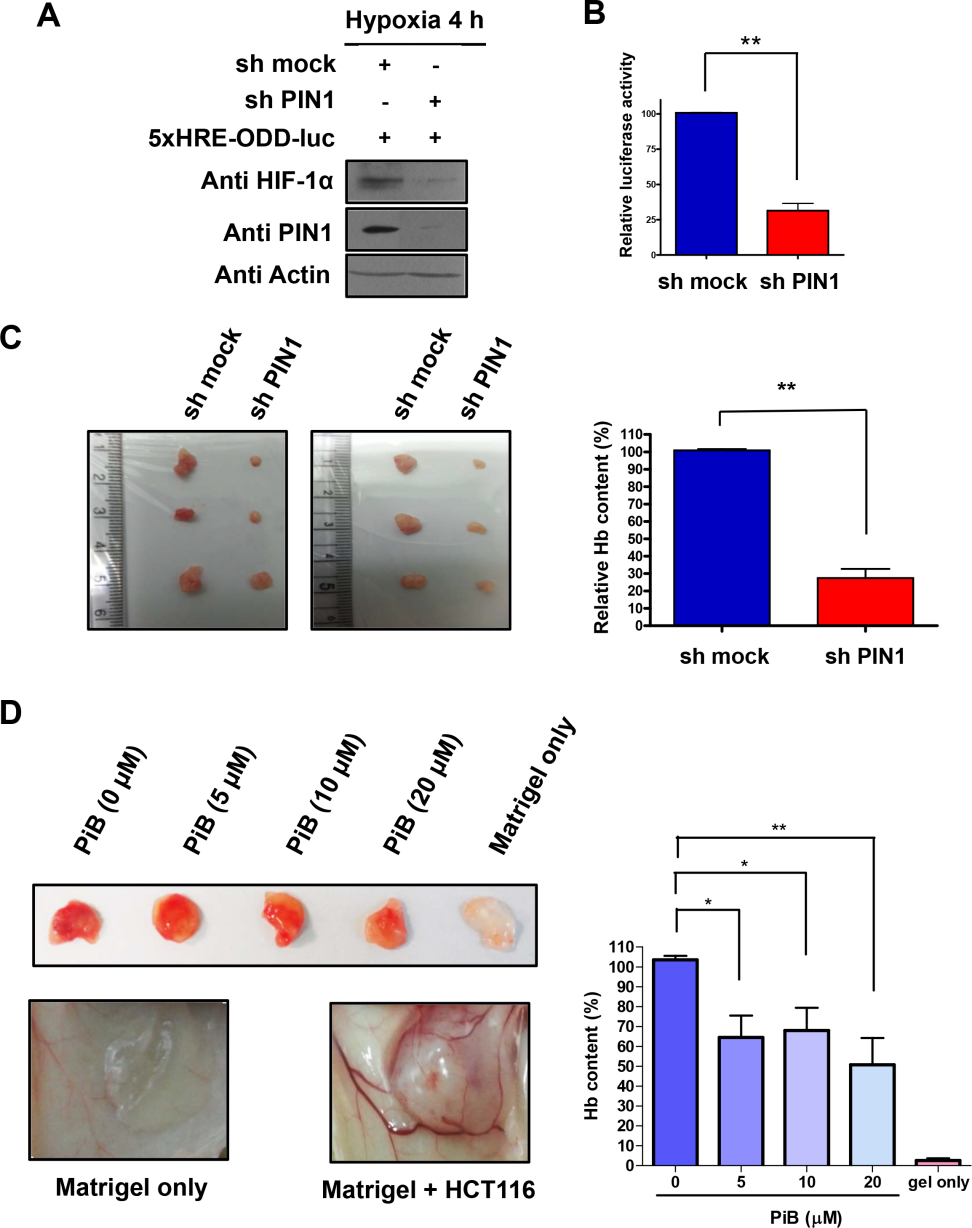
PIN1 enhances tumor angiogenesis *in vivo*. A) Silencing of PIN1 via sh-RNA lentiviral vector reduces the protein levels of HIF-1α in HCT116/5xHRE-ODD-luc cells. Western blot analysis was performed with 50 μg total cell lysate form HCT116/5xHRE-ODD-luc cells infected with control or sh-PIN1 virus. B) The luciferase activity of HRE in HCT116/5xHRE-ODD-luc cells infected with sh-PIN1 virus was reduced compared to control cells. C) HCT116/5xHRE-ODD-luc cells were infected with sh-PIN1 or control virus. The cells were subcutaneously injected into male BALB/c nu/nu mice (n = 6). Hb levels of Matrigel plugs from excised tumors after autopsy. Hb levels in the supernatant were quantified with the Drabkin’s reagent kit. The data are means ± SD from replicate experiments (n = 6) (**P < 0.001). D) PiB inhibited tumor angiogenesis in nude mice. Growth factor-reduced BD Matrigel containing HCT116 cells and indicated concentrations of PiB were subcutaneously injected into male BALB/c nu/nu mice as described in Materials and Methods. The photos are representative Matrigel plugs. Hb levels of Matrigel plugs. Hb levels in the supernatant were quantified with the Drabkin’s reagent kit. The data are means ± SD from replicate experiments (n = 4) (*P < 0.05, **P < 0.001).

To further assess the role of PIN1 in angiogenesis in an animal model, we conducted a Matrigel plug assay in nude mice. For this purpose, HCT116/5xHRE-ODD-luc cells were infected with control or sh-PIN1 lentiviruses. The expression of HIF-1α in cells infected sh-PIN1 virus was reduced than that of control cells (**Fig 7A**). Moreover, cells infected with sh-PIN1virus showed weak HIF1 reporter luciferase activity compared to control cells infected with control virus (**Fig 7B**). After verifying the stable expression of sh-PIN1 in the HCT116/5xHRE-ODD-luc cells, the cells were mixed with Matrigel and subcutaneously injected into male BALB/c nu/nu mice. The Hb levels of Matrigel plugs from excised tumors after autopsy were measured as described in Materials and Methods. Hb content in the Matrigel plugs from tumors of mice injected with cells harbouring sh-PIN1 lentivirus was significantly lower than that from mice injected with sh-mock virus infected control cells (**Fig 7C**). In another study, Matrigel plugs containing HCT116 and three different doses of PiB were injected subcutaneously in mice, and the degree of vascularization into Matrigel plugs was evaluated after 10 days. Macroscopic analysis of Matrigel plugs in mice without PiB treatment showed intense vascularization, whereas the Matrigel implant in mice treated with PiB displayed weaker angiogenesis. Hb contents in the plug were determined as a relative angiogenesis index. The angiogenic response observed by macroscopic analysis was consistent with quantitative results obtained by measuring the Hb levels in the Matrigel. Hb content in the Matrigel plugs in mice treated with PiB was significantly lower. (**Fig 7D**).

### PIN1 inhibition reduces transcriptional activation of HIF-1 in tumor hypoxia as determined by *in vivo* bioluminescence imaging

**Fig 8:**
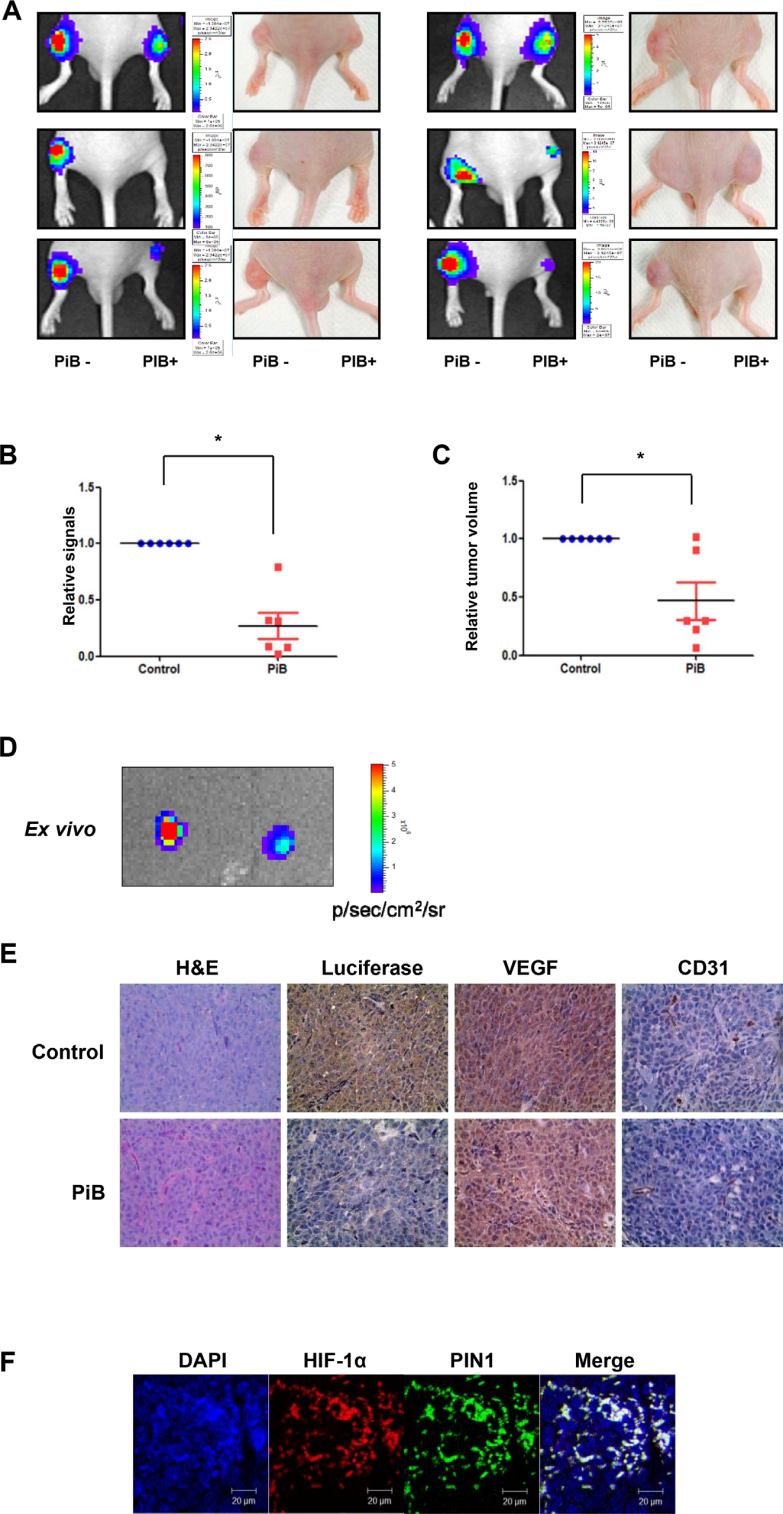
PiB reduces transcriptional activation in tumor hypoxia *in vivo* as assessed by bioluminescence imaging. A) *In vivo* bioluminescence imaging. Luciferase signals from PiB-treated tumor were lower than those from non-treated tumor. B) From ROIs analysis of *in vivo* bioluminescence imaging, signals in tumors from PiB treated mice were significantly reduced compare to non-treated tumor (P = 0.0016). C) The size of PiB-treated tumor was significantly decreased (P = 0.0212) compared to non-treated tumors. D) Bioluminescence images from excised tumors after autopsy showed that luciferase signals in tumor tissues from PiB-treated animals were lower than that of control tumor, indicating reduced activation of HRE. E) Immunohistochemistry data showed that tumors from PiB-treated mice express less luciferase, VEGF, and CD31 compared to non-treated tumors. F) Co-localization of PIN1 and HIF-1α was observed using confocal fluorescence microscopy. Scale bar, 20 μm.

**Fig 9:**
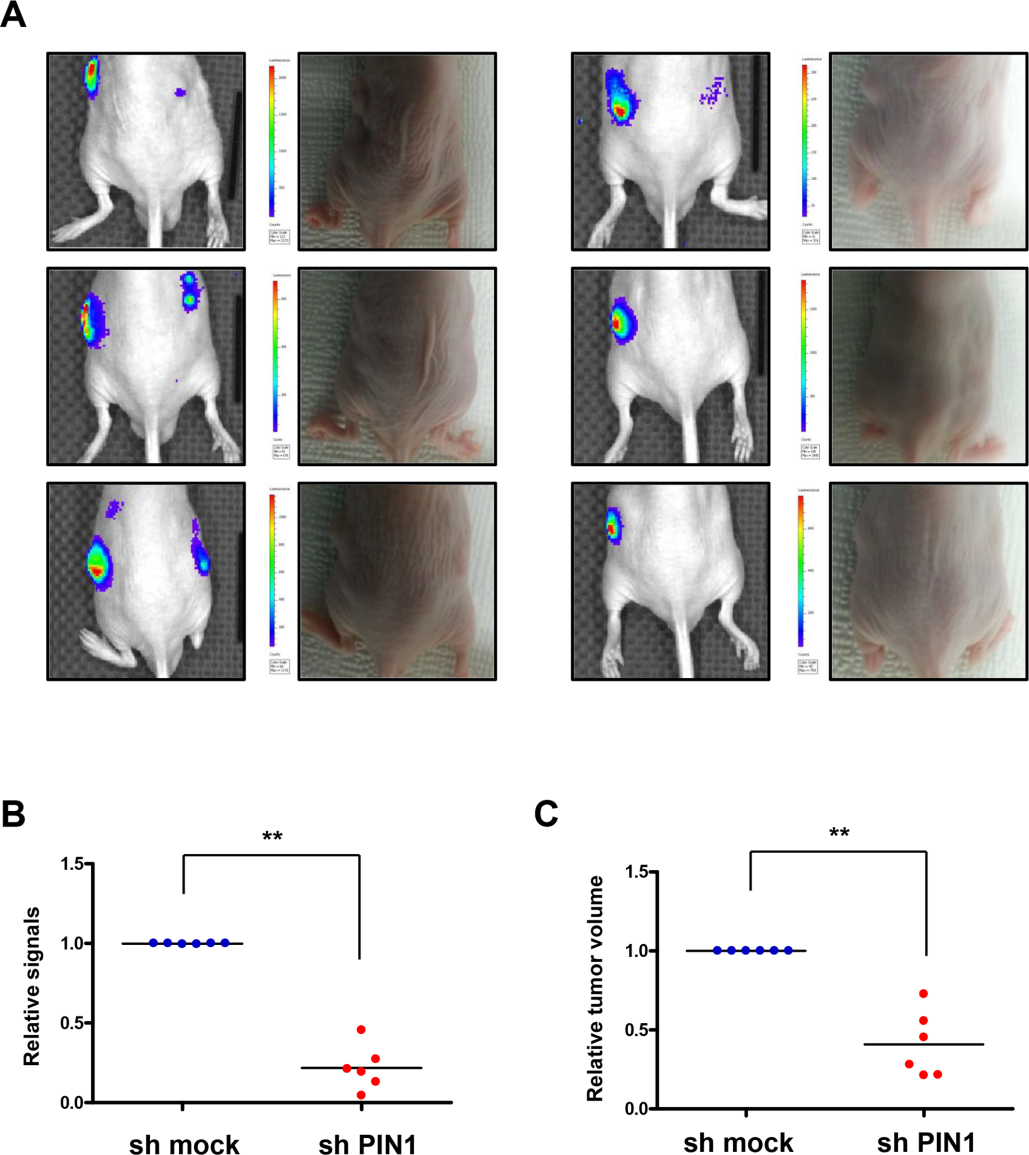
Silencing of PIN1 reduces transcriptional activation in tumor hypoxia *in vivo* as assessed by bioluminescence imaging. A) *In vivo* bioluminescence imaging. HCT116/5xHRE-ODD-luc cells were infected with sh-PIN1 or control virus. The cells were subcutaneously injected into male BALB/c nu/nu mice (n = 6). Luciferase signals from sh-PIN1 infected tumor were lower lower than those from sh-mock infected tumor. B) Signals in tumors infected sh-PIN1 were significantly reduced compare to tumors of sh-mock from ROIs analysis of *in vivo* bioluminescence imaging. C) The volume of tumors infected sh-PIN1 was significantly decreased compare to control tumors.

Bioluminescence imaging and tumor size measurement were performed in a murine colon cancer xenograft model in which the luciferase protein that responds to the tumor hypoxia is expressed, and visible light is produced for a fixed period of time after administration of the luciferin substrate. Luciferase signals from tumors from PiB-treated mice were lower than those from vehicle-treated animals (**Fig 8A**). From ROIs analysis of *in vivo* bioluminescence imaging, signals in the tumors from PiB treated mice significantly reduced (P = 0.0016) (**Fig 8B**). The size of tumors was significantly decreased by PiB treatment (P = 0.0212) (**Fig 8C**). Bioluminescence images from excised tumors after autopsy showed that luciferase signals in tumor tissues from PiB-treated mice were much lower, indicating reduced activation of HIF-1α (**Fig 8D**). Immunohistochemistry data showed that PiB treatment resulted in less luciferase activity and expression of angiogenesis-related proteins, such as VEGF and CD31 (**Fig 8E**). Co-localization of PIN1 and HIF-1α was verified by confocal fluorescence microscopy (**Fig 8F**). Consistent with PiB treatment, luciferase signals from sh-PIN1virus infected tumors were lower than those from sh-mock virus infected tumor (**Fig 9A**). Signals in tumors infected with sh-PIN1 virus were significantly reduced compared to those from sh-mock infected tumors based on ROIs analysis of *in vivo* bioluminescence imaging (**Fig 9B**). Likewise, the volume of tumors infected with sh-PIN1 was significantly smaller compared to sh-mock control tumors (**Fig 9C**).

## Discussion

Though PIN1 is overexpressed in many human tumors including those formed in prostate, breast, lung, liver, and colon [18, 25–27], some investigators have suggested the tumor suppressor function of PIN1 [28]. Thus, the role of PIN1 in tumorigenesis is still controversal. PIN1 is the peptidyl-prolyl cis/trans isomerase that binds and isomerizes specific phosphorylated Ser/Thr-Pro motifs in a subset of proteins, including those encoded by oncogenes or tumor suppressor genes [29, 30], thereby altering their structures and functions. In a variety of tumors, HIF-1α is overexpressed and modulates tumor cell growth, metastasis, and angiogenesis [31–33]. Interestingly, HIF-1α and PIN1 have many common interaction partners including p53 [34], NF-κB [35], cyclin D1 [36], and β-catenin [37]. However, the interaction between HIF-1α and PIN1 was not fully clarified. Therefore, it will be interesting to determine whether there is association between the prevalent PIN1 overexpression and constitutive HIF-1α activation in various tumors and cancerous cells.

Our present study demonstrated an oncogenic role for PIN1 in enhancing the stability of HIF-1α through its prolyl-isomerase activity. PIN1 interacted directly with phosphorylated HIF-1α and increased the stability of HIF-1α by inducing a conformational change of this transcription factor. Recently, Jalouli et al. reported that PIN1, by inducing a conformational change in HIF-1α, upregulated the transcriptional activity of HIF-1α without influencing its stability [24]. On the contrary, we have revealed that PIN1 directly binds to HIF-1α and enhances its stability as well as transcriptional activity. We showed that both genetic and pharmacologic inhibition of PIN1 accelerated a degradation of HIF-1α in hypoxic conditions. The phosphorylation of HIF-1α at Ser^641^/Ser^643^ is considered to be essential for its interaction with PIN1 [24]. Recently, it has been reported that phosphorylation of Ser^668^ of HIF-1α stabilizes it by CDK1 [12]. Interestingly, Ser^668^ is a putative PIN1 binding site. Thus, stabilization of HIF-1α is likely to involve multiple phosphorylation sites. Though our study reveals the increased stability of HIF-1α induced by PIN1, Lonati et al. have shown that PIN1 promotes degradation of HIF-1α via GSK3β in Alzheimer’s disease (AD) [38]. Inverse association between AD and cancer has been reported [39]. Patients with AD show a reduced risk of cancer, while cancer survivors are less likely to develop AD. A recent study provided new insights into the opposite role of PIN1 in the pathogenesis of cancer and AD [40].

In order to grow beyond a certain size, tumors need a dedicated vascular network for the sufficient supply of oxygen and essential nutrients [41]. Hypoxia stimulates the tumor angiogenesis, an important hallmark of cancer progression [42] through up-regulation of VEGF and other angiogenic cytokines. In tumor hypoxia, HIF-1α is an important regulator of the angiogenesis [43]. Our finding is consistent with previous studies in that PIN1 regulates HIF activity and VEGF expression [24]. In this study, the pharmacologic inhibition of PIN1 with PiB inhibited HIF-1α-mediated VEGF expression, and angiogenesis. The antiangiogenic activity of PiB may account for its capability to inhibit tumor growth in a xenograft model.

While our manuscript was in preparation, All-*trans* retinoic acid (ATRA) was reported as a new PIN1 inhibitor. ATRA binds to the PIN1 active site and facilitates PIN1 degradation, resulting in suppression of acute promyelocytic leukemia cell growth [44]. ATRA inhibits PIN1 expression as well as its catalytic activity, while PiB inhibits only catalytic activity. Notably, PIN1 phosphorylation on Ser^16^ was elevated upon PiB treatment. We speculate that Ser^16^ phopshorylation of PIN1 diminishes its substrate binding activity. Oxygen glucose deprivation was found to inhibit catalytic activity of PIN1 through protein kinase A-mediated Ser^16^ phosphorylation [38], lending support to our speculation [17]. In future studies, it will be worthwhile determining whether PiB phosphorylates PIN1^S16^.

Besides its regulation through direct interaction with PIN1, HIF-1α can be indirectly regulated via the tumor-suppressor protein promyelocytic leukemia (PML). PML is degraded by KLHL20, leading to enhanced accumulation of HIF-1α [45]. PIN1 was found to be involved in degradation of PML, consequently inducing HIF-1α upregulation [46]. Additional studies are required to determine whether PIN1 can indirectly regulate HIF-1α stability by stimulating the degradation of PML.

Tumor hypoxia is a dynamic phenomenon in cancer. So it is necessary to precisely measure the HIF-1 activity in tumor microenvironment. Bioluminescence imaging techniques could enable visualization of dynamic changes of tumor hypoxia *in vivo* [47]. By employing the bioluminescence assay, we were able to demonstrate the suppression of HIF-1α transcriptional activation by pharmacologic inhibition of PIN1 in tumor hypoxia.

The phosphorylation of Ser/Thr-Pro motifs by Pro-directed kinases has a key role in controlling cell division [48]. PIN1 has been considered to be critical in regulating mitotic progression through its phosphorylation-dependent proline isomerase activity. PIN1 catalyzes changes in the conformation of cell cycle regulatory proteins, such as p53 [49], p27Kip1 [50], Cdc25 [51], and Rb [52]. According to our present study and a previous investigation by others, PIN1 causes a conformational change in HIF-1α and enhances HIF-1 activity. In fact, PIN1 is a direct target of E2 transcription factor 1 (E2F1) [53], which plays a pivotal role in regulation of cell cycle progression from G1/S [54]. The consequence of increased phosphorylation of Rb is the release of Rb-bound E2F1, which is known to induce tumor angiogenesis [55]. Recently, it has been reported that PIN1 inhibits Rb dephosphorylation in regulation of cell cycle [52], and HIF-1 stimulates angiogenesis through cooperation with Rb [56]. Therefore, these studies show that PIN1 could also change conformation of IFHIF-1α which may stimulate angiogenesis in cooperation with modulation of the cell cycle related protein, Rb.

Taken together, PIN1 is a potent modulator of phosphorylated HIF-1α in inducing angiogenesis, presumably by regulating the conformation of the phosphorylated Ser-Pro motifs in HIF-1α. These results suggest PIN1 as an attractive therapeutic target, inhibition of which could potentially destabilize HIF-1α to retard the angiogenesis. Furthermore, it is likely that further studies will be necessary to validate the clinical application of inhibitors of PIN1 in the management of cancer.

## Supporting Information

S1 Table Comparison of the length, the width, and the volume of tumors between control and PiB-treated mice. (DOCX)

S2 Table Comparison of the body weight changes in xenograft mice. (DOCX)
