# Correction: Peptidyl Prolyl Isomerase PIN1 Directly Binds to and Stabilizes Hypoxia-Inducible Factor-1α

**DOI:** 10.1371/journal.pone.0151517

**Published:** 2016-03-10

**Authors:** The PLOS ONE Staff

There are errors in Fig 1, the Fig 1 legend, and Fig 4. Please see the correct Fig 1, Fig 4 and their legends here. The publisher apologizes for the errors.

**Fig 1:**
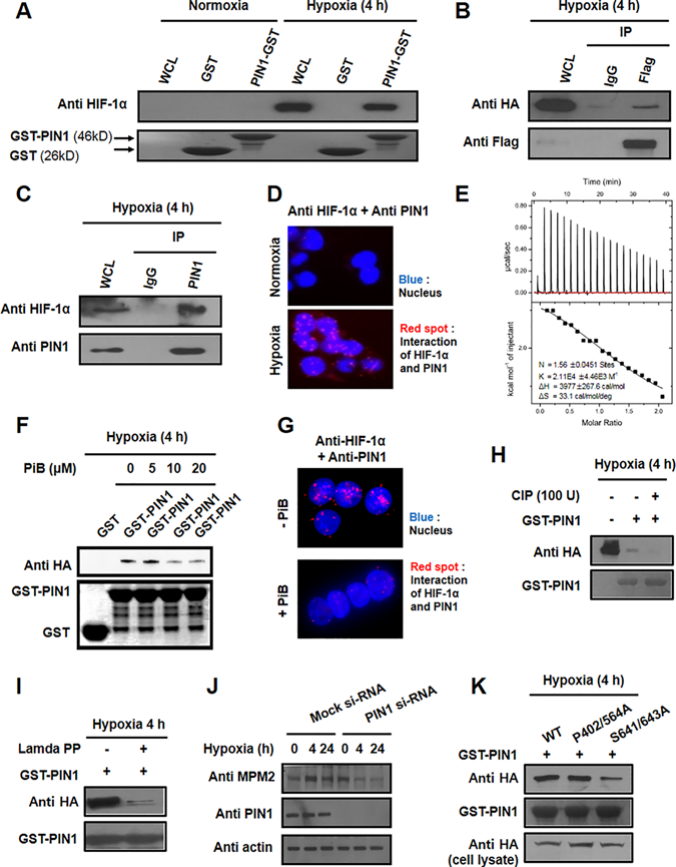
PIN1 physically interacts with HIF-1α in a phosphorylation-dependent manner. A) *In vitro* GST pull down assay. The cell lysates were incubated with GST or GST-PIN1 fusion protein, followed by addition of the GST beads. The precipitates were immunoblotted with anti-HIF-1α to show the bound HIF-1α and reprobed with anti-GST to show the precipitated GST and GST-PIN1. B) Interaction of HIF-1α and PIN1 in HCT116 cells. Cells were transfected with Flag-tagged HIF-1α and HA-PIN1, and stimulated with hypoxia for 4 h. The cell lysates were immunoprecipitated with anti-Flag antibody, and the precipitates were fractionated by SDS-polyacrylamide gel electrophoresis and blotted with anti-HA antibody. C) Association of endogenous PIN1 and HIF-1α. HCT116 cells were subjected to hypoxia for 4 h. Cell lysates were incubated with either normal IgG or anti-PIN1 as labeled and blotted with anti HIF-1α. D) Binding of HIF-1α and PIN1 *in situ*. The HCT116 cells were incubated under normoxia or hypoxia. Interaction of HIF-1α and PIN1 was visualized by Duolink analysis. HIF-1α and PIN1 were co-labeled with antibodies. Nuclei were counter stained with DAPI (blue). Scale bar, 20 μm. E) ITC indicates that PiB binds PIN1. Heat evolution as a function of adding increasing amounts of PiB to GST-PIN1. The heats of dilution were measured separately, and found to be <1% of the signal at the start of titration. Fitting the ITC data with Microcal analysis launcher software indicates that PiB binds to GST-PIN1. Values shown on the figure are from the fit of the displayed dataset. K = 2.11E4 ± 4.46E3M^-1^, N = 1.56 ± 0.0451 sites, ΔH = 3.977 ± 0.2676 Kcal/mol, ΔS = 33.1 cal/mol/deg. F) *In vitro* GST pull down assay. The cell lysates were incubated with GST or GST-PIN1 fusion protein, followed by treating with PiB and addition of the GST beads. The precipitates were immunoblotted with anti-HIF-1α to show the bound HIF-1α and reprobed with anti-GST to show the precipitated GST and GST-PIN1. G) Binding of HIF-1α and inactivated PIN1 *in situ*. HCT116 cells were incubated with or without PiB (20 μM). Interaction of HIF-1α and inactivated PIN1 was visualized by Duolink analysis. HIF-1α and PIN1 were co-labeled with antibodies. Nuclei were counter stained with DAPI (blue). H) CIP (50 U) was added to the supernatants at 30°C for the indicated time periods. Following incubation, GST or GST-PIN1 proteins were incubated with the supernatants for 4 h and then were pulled down with GST beads. Following incubation, reactions were stopped by the addition of SDS sample buffer, followed by SDS-PAGE. I) HA-HIF-1α proteins were purified using a commercially available kit. The proteins were treated with or without lamda phosphatase for 1 h at 30°C. Then GST-PIN1 proteins were incubated with purified HA-HIF-1α proteins and were pulled down with GST beads. The proteins were resolved in SDS-polyacrylamide gels and detected by immunoblotting. J) Cells were transfected with scrambled siRNA as a negative control or PIN1-siRNA for 72 h and treated with hypoxia for 4 h. Cell lysates were incubated with either normal IgG or anti-MPM2 as labeled and blotted with anti-PIN1. K) HA-HIF-1α, HA-HIF-1α^S402/564A^, and HA- HIF-1α^S641/643A^ were transfected in HCT116 cells. Whole cell extracts of HCT116 cells were prepared for pull down assays with GST-PIN1 proteins. The pull downed fractions were subjected with anti-HA antibody.

**Fig 4:**
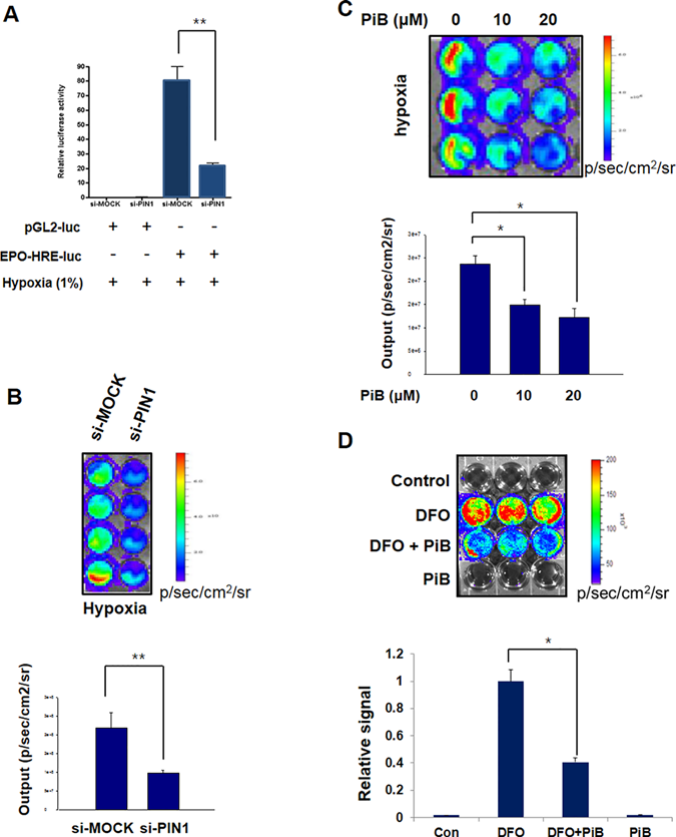
PIN1 regulates the hypoxia induced transcriptional activity of HIF-1. A) Effect of PIN1 knockdown on EPO-HRE-luciferase reporter activity under normoxia or hypoxia for 24 h. HCT116 cells were transfected with control or PIN1 si-RNA for 72 h and then were transfected with pGL2-luc and EPO-HRE-luc for another 24 h. Cells were lysed to analyze luciferase activities, which were normalized against β-galactosidase activities. B, C) *In vitro* HIF-1α bioluminescence assay. 3 x 10^5^ HCT116/5xHRE-ODD-luc cells were transfected with control or PIN1 si-RNA for 24 h (B) or treated with PiB (C) and harvested 8 h after treatment. The cells were incubated in each well of a 96-well-dish in a hypoxia chamber (1% O_2_) for 4 h before the medium was removed, washed and replaced with 1 ml PBS. Immediately after 100 μl luciferin was added into each well, ROIs were acquired with an array of exposure times (1, 30, 60, and 180 s). D) PIN1 regulates transcriptional activity of HIF-1 during hypoxia-mimic conditions. Hypoxia was induced by DFO (400 μM). HCT116/5xHRE-luc cells were treated with DFO only, DFO plus PiB (20 μM), or PiB (20 μM) alone for 8 h. ROIs were analysed by bioluminescence imaging from HCT116/5xHRE-luc cells after various treatments.

There is an error in the last sentence of the penultimate paragraph of the Discussion. The correct sentence is: Therefore, these studies show that PIN1 could also change conformation of HIF-1α which may stimulate angiogenesis in cooperation with modulation of the cell cycle related protein, Rb.
