# Supplementary material for: Peptidyl Prolyl Isomerase PIN1 Directly Binds to and Stabilizes Hypoxia-Inducible Factor-1α
Source: PLoS One. 2016 Jan 19;11(1):e0147038. doi: 10.1371/journal.pone.0147038 (PMC4718546; doi:10.1371/journal.pone.0147038)
Supplement: S1 Table — (DOCX) [file pone.0147038.s001.docx]

**S1 Table**. Comparison of the length, the width, and the volume of tumors between control and PiB-treated mice

|  | **Control tumor** | | | **PiB treated tumor** | | |
| --- | --- | --- | --- | --- | --- | --- |
|  | **Length (mm)** | **Width (mm)** | **L*(W/2)^2*3.14**  **(mm^3^)** | **Length (mm)** | **Width (mm)** | **L*(W/2)^2*3.14**  **(mm^3^)** |
| **#1** | 7.4 | 6.86 | 273.3692164 | 7.77 | 6.75 | 277.9061906 |
| **#2** | 8.53 | 6.6 | 291.679938 | 3.68 | 2.55 | 18.784422 |
| **#3** | 9.16 | 6.05 | 263.1939365 | 6.12 | 3.5 | 58.85145 |
| **#4** | 8.57 | 7.38 | 366.4065278 | 8.48 | 7.07 | 332.7394823 |
| **#5** | 17.91 | 13.74 | 2654.230944 | 10.48 | 9.79 | 788.4902419 |
| **#6** | 9.03 | 8.8 | 548.937312 | 6.61 | 5.63 | 164.4704596 |
