# Supplementary material for: Peptidyl Prolyl Isomerase PIN1 Directly Binds to and Stabilizes Hypoxia-Inducible Factor-1α
Source: PLoS One. 2016 Jan 19;11(1):e0147038. doi: 10.1371/journal.pone.0147038 (PMC4718546; doi:10.1371/journal.pone.0147038)
Supplement: S2 Table — (DOCX) [file pone.0147038.s002.docx]

**S2 Table**. Comparison of the body weight changes in xenograft mice

| **Mouse body weight (g)** | | |
| --- | --- | --- |
|  | **0 day** | **16 day** |
| **#1** | 19.5 | 18.7 |
| **#2** | 20.9 | 21.4 |
| **#3** | 20 | 19.7 |
| **#4** | 20 | 19.6 |
| **#5** | 23.2 | 21.7 |
| **#6** | 21.1 | 21.1 |
